# Enrichment of rare alleles within epigenetic chromatin marks in the first intron

**DOI:** 10.5808/GI.2019.17.1.e9

**Published:** 2019-03-31

**Authors:** Shin-Sang Jo, Sun Shim Choi

**Affiliations:** Division of Biomedical Convergence, College of Biomedical Science, Institute of Bioscience & Biotechnology, Kangwon National University, Chuncheon 24341, Korea

**Keywords:** first intron, minor allele frequency, promoter, rare allele

## Abstract

In previous studies, we demonstrated that some sites in the first intron likely regulate gene expression. In the present work, we sought to further confirm the functional relevance of first intron sites by estimating the quantity of rare alleles in the first intron. A basic hypothesis posited herein is that genomic regions carrying more functionally important sites will have a higher proportion of rare alleles. We estimated the proportions of rare single nucleotide polymorphisms with a minor allele frequency < 0.01 located in several histone marks in the first introns of various genes, and compared them with those in other introns and those in 2-kb upstream regions. As expected, rare alleles were found to be significantly enriched in most of the regulatory sites located in the first introns. Meanwhile, transcription factor binding sites were significantly more enriched in the 2-kb upstream regions (i.e., the regions of putative promoters of genes) than in the first introns. These results strongly support our proposal that the first intron sites of genes may have important regulatory functions in gene expression independent of promoters.

## Introduction

All eukaryotic genomes contain introns within their gene structures [1,2]. It has long been a mystery how cells have maintained such a seemingly burdensome entity over the time scale of evolutionary processes [3-8]. Recent high-throughput genomic technologies based on next-generation sequencing have helped to prove that intron sites are in fact subject to natural selection. In other words, introns play functional roles in various aspects of molecular dynamics occurring inside cells, such as regulation of gene expression, primary transcript splicing, and mRNA stability [9].

We have previously reported some evidence on the functional roles of introns—more specifically, the first ordinally positioned introns (referred to as first introns)—in regulating gene expression [10,11]. Briefly, we investigated whether first introns were conserved to a significantly greater extent than other downstream introns [10]. In addition, we showed that the greater conservation of first introns, associated with a greater number of epigenetic marks, was independent of the conservation of the 5' flanking or promoter regions of genes [10]. In other words, the signatures that we found in the first introns were not a spillover effect of the promoter or 5′ flanking regions of the genes. Moreover, we expanded our first study on introns by adding more multi-omics data, coupled with the enrichment of epigenetic marks in the first introns [11]. In that study, we showed that trait-associated single-nucleotide polymorphisms (TASs) were significantly more enriched in first introns than would be expected by random chance. Second, we showed that patterns of epigenetic signals varied by expression specificity or gene essentiality. Third, we expanded the depth of our analysis to show that genes carrying multiple first-intron TASs constituted a large protein-protein interaction network with each other, ultimately connecting to the *UBC* protein, which plays a well-established role in ubiquitination.

Several other recent reports have confirmed the functional importance of introns, particularly first introns, in regulating gene expression. For instance, the first intron of the *Col1A1* gene in mice was found to play a role in tissue-specific and developmental gene expression [12]. Similarly, the first introns of *PRF1* and *PRF2* in *Arabidopsis thaliana* were observed to affect constitutive gene expression in vegetative tissues [13]. The determination of the transcription start site of the *UBC* gene was found to be dependent on the presence of its first intron [14].

In the present work, we sought to provide further evidence on the functionality of first introns, by investigating whether rare alleles located in the sites overlaying epigenomic marks in first introns were more enriched than in 2-kb upstream sequences and other downstream introns.

## Methods

### Obtaining genomic structures, including introns and promoters, in the human genome

We retrieved information about introns and other genomic structures using the same methods as in our previous studies [10,11]. Briefly, the 300-bp regions around exon-intron boundaries were excluded to avoid including splicing control sites [15]. Extremely short or long genes were also removed; too short was defined as a total length < 1 kb, while too long was defined as a length > [third quartile + (interquartile range × 1.5)] of the total length. Sequences from the Y chromosome were removed. Repeats were removed using RepeatMasker (http://repeatmasker.org/cgi-bin/WEBRepeatMasker). Repeat sequences in the 2-kb upstream sequences were also masked using RepeatMasker after they were retrieved from the University of California, Santa Cruz (UCSC) genome browser.

### Rare single-nucleotide polymorphisms

dbSNP152 was downloaded from the dbSNP database through ftp://ftp.ncbi.nih.gov/snp/ to obtain information about rare single-nucleotide polymorphisms (SNPs). Rare SNPs were retrieved after excluding indels, SNPs with a minor allele frequency (MAF) of > 0.01, and SNPs without information about the MAF. The selected rare SNPs were mapped onto the intronic sequences filtered by the criteria described above. A total of 4,128,907 rare SNPs were mapped onto the filtered introns, of which 1,417,251 (34%) were mapped onto the first introns, whereas 505,637 SNPs were mapped onto the 2-kb upstream regions of genes with repeats masked.

### Regulatory chromatin marks

Regulatory signals, such as DNaseI hypersensitive sites (DHSs), CCCTC-binding factor (CTCF) binding sites, transcription factor binding sites (TFBSs), and other regulatory chromatin marks including H3K4me1, H3K4me3, H3K9me3, H3K27ac, and H3K27me3 were downloaded from the ENCODE browser (https://www.encodeproject.org), with the search limited to ENCODE Tier-1 cell lines, including GM12878, H1-hESC, and K562 cells. The site information about all these regulatory signals was mapped onto the intron sequences and the 2-kb upstream sequences, as described above.

### Statistical tests

All statistical tests and box plot representations were conducted using R (version 3.5.2) [16] with R studio (version 1.1.463) [17]. The Wilcoxon test was performed using the *stat_compare_means* function of the *ggpubr* R package (https://rpkgs.datanovia.com/ggpubr/index.html) to investigate the statistical significance of 6 differences among groups. In-house Python scripts were used for the remaining mapping and parsing analyses (version 3.7.2, http://www.python.org).

## Results

### The first introns and the 2-kb upstream regions showed different enrichment patterns of epigenetic signals

**Fig. 1.**
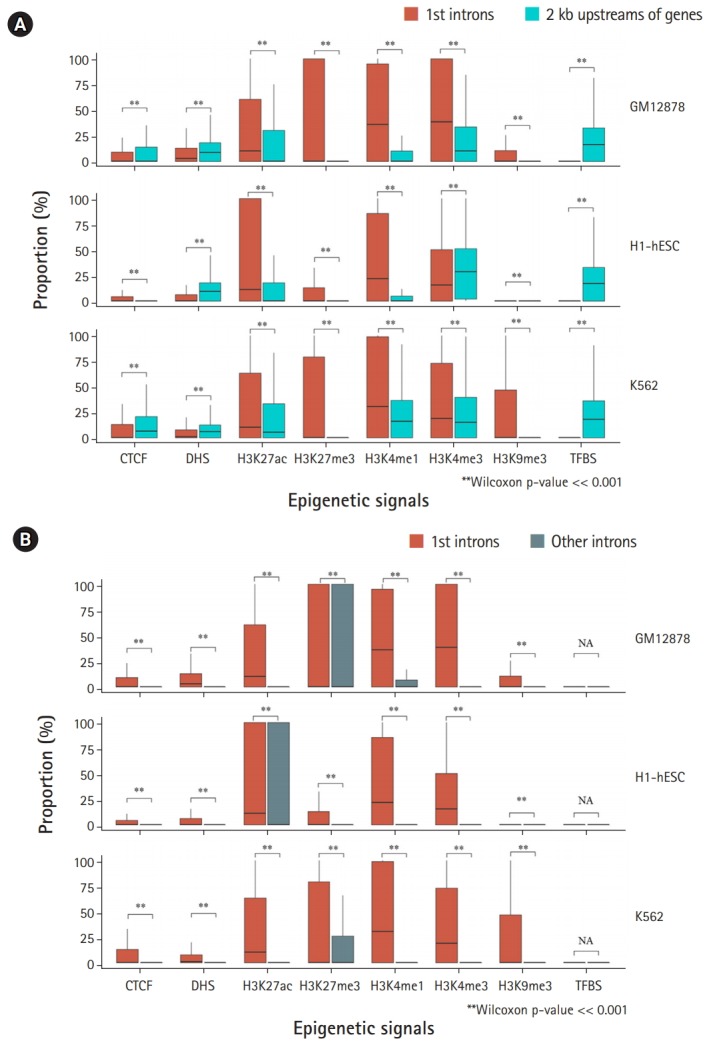
Comparison of the proportions of rare alleles in the first introns, in the 2-kb upstream regions, and in other downstream introns. (A) The proportions of rare alleles assigned to each chromatin mark located in the first introns and the 2-kb upstream regions of genes were estimated and plotted using the box plot method (see Methods). (B) The proportions of rare alleles assigned to each chromatin mark located in the first introns and other downstream introns. CTCF, CCCTC-binding factor; DHS, DNaseI hypersensitive site; TFBS, transcription factor binding site; N/A, not available. **Statistical significance estimated by a p-value much less than 0.001 from the Wilcoxon rank-sum test.

It is a well-established principle that mutant alleles generated from functionally important sites show very low population-level frequencies. In other words, a MAF < 0.01 in the population, which is a common definition of a rare allele, is a good indicator of the functional importance of an allele. Therefore, we hypothesized that the number of sites with an MAF < 0.01 (i.e., the proportion of rare alleles) per unit of genomic length should be higher if a genomic region carries a greater quantity of functionally important sites. Under this hypothesis, we investigated differences in the distribution of the patterns with which rare alleles were assigned to epigenetic regulatory marks in the first introns and the 2-kb upstream region of genes. Interestingly, we found that most epigenetic signals located in the first introns carried a significantly greater proportion of rare alleles than those in the 2-kb upstream region, whereas the proportions of rare alleles assigned to CTCFs, DHSs, and TFBSs were more enriched in the 2-kb upstream region than in the first introns (Fig. 1A). However, it should be noted that the higher proportion of rare alleles in the first intron than in the 2-kb upstream regions was inconsistent for H3K4me3 signals across different cell types. These results may imply that the roles of epigenetic signals in the first introns and the 2-kb upstream region might be independent or complementary to each other. The three different Tier-1 cell lines yielded essentially the same results, although there were some slight differences among the cell lines.

### Rare alleles are more enriched in the epigenetic signals located in the first introns than in other introns

We next investigated whether the proportions of rare alleles in the first introns were significantly different from those in other downstream introns. As expected, rare alleles in the first introns were significantly more enriched with all the epigenetic signals we tested than other downstream introns (Fig. 1B). It seems reasonable that the first introns carried significantly more rare alleles than the other downstream introns at the sites where epigenetic chromatin marks are located, considering that first introns tend to harbor a greater quantity of epigenetic signals [10,11]. A further notable finding is that the different Tier-1 cell lines showed significantly different amounts of rare alleles in H3K27ac, an active chromatin mark, and in H3K27me3, a polycomb repressive mark. It seems that embryonic stem cells (i.e., H1- hESC cells) tended to carry more rare alleles in active signals (H3K27ac) than in repressive signals (H3K27me3), whereas fully differentiated cells, such as GM12878 and K562 cells, showed higher proportions of rare alleles in repressive marks than in active marks (Fig. 1B). However, the observation of a significantly greater quantity of rare alleles in the first introns than in other introns still stands, despite these differences in the proportions of rare alleles in each chromatin mark site in different cell types.

## Discussion

In our previous research, we demonstrated that the first intron is special compared with other introns for the following reasons: it is the longest intron; it has the largest proportion of conserved sites among introns; it harbors the highest proportion of TASs among introns; and it is the most enriched with various chromatin regulatory signals. Moreover, chromatin regulatory signals are significantly linked to the level and breadth of gene expression. In the 8 present study, we added further evidence, proving that the first intron carries functional sequences (i.e., sequences for locating various active or repressive epigenomic marks) by analyzing the proportions of rare alleles in the chromatin marks located within introns.

Unfortunately, the influence exerted by intronic sequences on the control of gene expression has yet to be experimentally proven. Experimental studies have demonstrated links between some intronic mutations occurring at the sites of splicing regulation or miRNA binding and certain diseases [18,19]. However, controversy remains regarding whether intronic alterations can directly cause changes in gene expression. According to Nott et al. (2003) [20], gene expression of the same coding genes was not affected by the absence or presence of introns. Moreover, some studies argued that intronic sequences evolve through neutral evolution due to the absence of selective constraints, and that their evolutionary rates are faster than fourfold degenerate sites [7,21].

The difficulty in experimentally proving the functions of intronic sequences in gene regulation seems to be mainly due to the complexity of the epigenomic chromatin signals that exist in different combinations in different cell or tissue types. Moreover, gene expression patterns are controlled by highly complex combinatorial interactions among these regulatory signals.

Although the functional importance of introns has not yet been experimentally proven, our findings provide support for the proposal that intron sites, particularly those in the first intron, are subject to natural selection. We suggest that regulation of gene expression is likely to ultimately emerge as a function of the sites implicated as being subject to natural selection.
